# Nano-scientific Application of Atomic Force Microscopy in Pathology: from Molecules to Tissues

**DOI:** 10.7150/ijms.41805

**Published:** 2020-03-15

**Authors:** Tony Mutiso Kiio, Soyeun Park

**Affiliations:** College of Pharmacy, Keimyung University, 1095 Dalgubeoldaero, Daegu 42601, Republic of Korea.

**Keywords:** atomic force microscopy, adhesion properties, disease diagnosis, morphology, mechanical compliance, molecular recognition

## Abstract

The advantages of atomic force microscopy (AFM) in biological research are its high imaging resolution, sensitivity, and ability to operate in physiological conditions. Over the past decades, rigorous studies have been performed to determine the potential applications of AFM techniques in disease diagnosis and prognosis. Many pathological conditions are accompanied by alterations in the morphology, adhesion properties, mechanical compliances, and molecular composition of cells and tissues. The accurate determination of such alterations can be utilized as a diagnostic and prognostic marker. Alteration in cell morphology represents changes in cell structure and membrane proteins induced by pathologic progression of diseases. Mechanical compliances are also modulated by the active rearrangements of cytoskeleton or extracellular matrix triggered by disease pathogenesis. In addition, adhesion is a critical step in the progression of many diseases including infectious and neurodegenerative diseases. Recent advances in AFM techniques have demonstrated their ability to obtain molecular composition as well as topographic information. The quantitative characterization of molecular alteration in biological specimens in terms of disease progression provides a new avenue to understand the underlying mechanisms of disease onset and progression. In this review, we have highlighted the application of diverse AFM techniques in pathological investigations.

## Introduction

**Figure 1:**
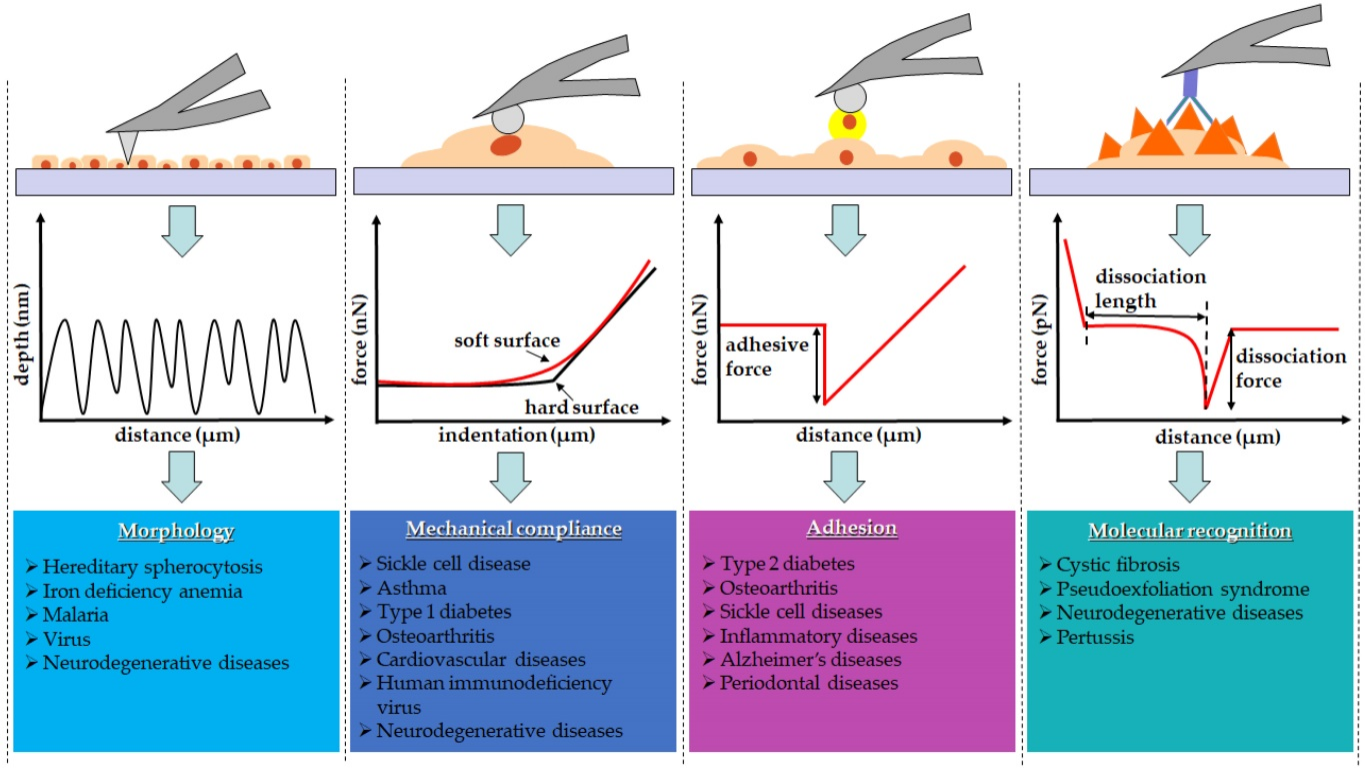
Various AFM-based techniques adapted to investigate biological specimens. The schematic drawings of an experimental technique and a corresponding line scan or force curve were illustrated for each experimental technique.

AFM has emerged as a powerful nanoscopic platform to investigate various biological systems due to its applicability in physiological conditions. Unlike other scanning probe microscopic tools, AFM can be operated under physiological conditions. In addition to its sub-nanometer resolution and pico-newton force sensitivity, AFM is capable of recognizing single molecular events, compositional changes, and intercellular interactions occurring in heterogeneous biological systems during disease progression (Fig. 1) 1,2. Many pioneer studies have investigated the possibility of utilizing AFM as a nano-diagnostic tool to establish unbiased quantitative assessment rubrics to monitor pathological conditions 3. Comparative studies between healthy and pathologic specimens were performed using AFM. Nanoscopic structures, mechanical properties, and single molecular events were directly observed in cells and tissues via a minimally invasive surgical intervention 4.

Diseases usually evolve as a result of unwanted morphological alterations. In order to develop therapeutic interventions, it is important to identify the causes of disease-related phenotypes. AFM is capable of revealing disease-related structural changes 5,6. It obtains topographic images based on the interatomic potential between the AFM probe and the samples. As the probe scans across the sample surface, the interatomic potentials move the cantilever in the perpendicular direction in order to keep a probe-sample interaction force constant. According to the cantilever's movement, the topography reflecting the contours of the sample surface is generated 7,8.

In addition, AFM as a nano-indenter is used to measure the mechanical compliances of biological samples. Cells actively reorganize their internal structure, leading to the alteration of their mechanical properties 9,10. Many studies reported changes in the mechanical compliances of cells and tissues as pathologic manifestation 11,12. The simple indentation of the AFM probe onto the sample yields a force-distance curve 13. By analyzing the obtained force-distance curve using mathematical models, the mechanical compliances, *i.e.*, elastic moduli, were accurately determined. The Hertz model has been the most widely utilized mathematical model for this purpose. The mathematical models modified from the Hertz model have also been adapted in order to resolve the issues raised by the heterogeneous nature of biological samples 14-16.

Cell adhesion is essential in biological processes including cell proliferation, migration, and fate 17. The adhesive strength of cells varies with substratum, topography, and chemo-mechanical properties surrounding the cellular microenvironment. Especially, the molecular composition and the mechanical properties of the extracellular matrix play a key role in cell spreading and migration 18. Adhesion force is characterized by AFM-based pull-off force measurements in which the AFM probe approached a surface and then subsequently retracted. The pull-off force is defined as the maximum attractive force during the retraction of the tip from the surface 19, 20. Pioneer studies have provided insight into disease-associated alterations in the cell-cell adhesion molecules expressed on the cell surfaces. For instance, the adhesion of ICAM-1, VCAM-1 and integrin VLA-4 on endothelial cells and monocytes is known to be a major contributor to determine the fate of inflammatory diseases 21-23. The microbial adhesion on the enamel surfaces causes periodontal infection such as dental caries and cavities 21. Moreover, metal ions such as zinc (II) and copper (II) are implicated with increased aggregation of the Aβ peptide into toxic oligomers, accelerating the pathogenesis of Alzheimer's disease 22,23. Thus, the AFM- based studies attempted to investigate whether the disruption of the cell-cell/substrate adhesions affects the progression of these diseases 24-28.

The quantitative characterization of intermolecular interaction is essential for a profound understanding of biological processes 29. AFM probe functionalized with antibodies were utilized to recognize the antigenic sites on the surface of the cell membrane 30. The extension lengths and the rupture forces were measured from the force-distance curves obtained while the probe retracted from the sample. They reflect the specific binding forces between the antibody attached on the AFM probe and the antigens on the cell surface. The map recognizing these specific binding forces refer to the molecular recognition imaging technique using the AFM. Using this technique, one can recognize antigenic sites on the cell membrane 31. A variety of biosystems have been investigated by simultaneously obtaining topography and molecular recognition image. Molecular interactions with high specificity including antigen-antibody, DNA aptamers, and ligand-receptor pairs have been utilized for this purpose 32,33. The AFM molecular recognition imaging techniques were utilized to investigate the pathologic conditions such as cystic fibrosis, pseudoexfoliation 34, cystic fibrosis 35, pertussis 36 and neurodegenerative diseases 37, which were known to involve alterations in the molecular composition of cell membrane.

Many diseases display numerous pathophysiological modifications including structural or compositional changes, which can be used as diagnostic or prognostic markers 38-41. AFM has been adapted to investigate such pathophysiological modifications to understand the underlying mechanism of various pathologic conditions. To do this, it is required to obtain biological specimens including blood samples and surgical specimens from patients and healthy volunteers in the clinical setting. For example, erythrocyte cells were isolated from blood samples in order to investigate hereditary spherocytosis, iron deficiency anemia, sickle cell disease, and type 2 diabetes using AFM 27,42-46. In addition, it is relatively easy to obtain tissue samples from asthma patients for AFM studies because bronchial tissues can be collected through routine bronchoscopy 47. However, studies on some diseases require more painful procedures for specimen collection. For instance, in order to investigate osteoarthritis, it is required to isolate chondrocyte cells from human articular chondrocytes tissues 48,49, and more rigorous surgical approach is required to obtain tissue samples from animals. Human tissues discarded from surgical procedures are also used in AFM studies. Islet tissue was surgically obtained from mice pancreas for AFM study on type 1 diabetes 50. Similarly, human lens harvested from patients during cataract surgery was used for AFM study on pseudoexfoliation syndrome 51.

In this review, we address the potential of AFM as a clinical diagnostic tool to detect the pathological changes associated with various diseases. So far, there have been advances in the use of AFM for cancer diagnosis and prognosis. In our previous publications, we addressed various aspects of AFM applications in cancer biology 52. However, in this review, we have focused on other diseases apart from cancer.

## Morphology

Since live erythrocytes were first imaged by AFM, there has been a remarkable breakthrough in cell imaging with AFM under physiological conditions 53. Many AFM studies have been performed on various kinds of cells under controlled pH and temperature in liquid environments, to avoid unfavorable distortion in images. AFM images provide detailed morphological features such as size, shape and surface topography at sub-nanometer resolutions 54-57. In addition, it provides information on cellular architectures such as structural, conformational, and constitutional information of cytoskeletal proteins and membrane lipids 58-61. During pathological progression, cells often undergo morphological modulations. Due to its high resolution, AFM can visualize early subtle changes in cell morphology prior to significant pathological conditions beyond the detection limit of other microscopic investigations. Morphological changes can also be observed with AFM shortly after therapeutic intervention, and thus, therapeutic efficacy can be evaluated by AFM images.

A healthy erythrocyte has a biconcave disk shape with a very shallow center. A disorder in erythrocytes can be detected by monitoring the shape, size, membrane proteins, number, and hemoglobin contents 62,63. Abnormalities found in erythrocytes are pathological indicators in many diseases such as hereditary spherocytosis (HS) 64, anemia 65, and malaria 66. Physicians clinically diagnose anemia by measuring the number of erythrocytes and the amount of ferritin — an iron-containing protein in the blood 67. Clinical guideline for the diagnosis of HS also recommends close monitoring of erythrocytes abnormality. To diagnose diseases associated with abnormal erythrocytes, hematocrit, osmotic fragility, and direct anti-globulin tests are usually carried out to measure the volume as well as the fragility of erythrocytes and determine the antibodies attached to erythrocytes 64,67. Nevertheless, the aforementioned tests lack specificity and often lead to false positive results, predicting a wide spectrum of clinical disorders 64,66-68.

Several studies suggested that AFM images can serve as a diagnostic alternative tool with higher specificity and accuracy as summarized in Table 1 54,55,69. HS is one of the hemolytic disorders caused by congenital defects. Anemia, jaundice, and splenomegaly are clinical symptoms experienced by HS patients. AFM study identified the morphological hallmark in HS patients as small spheroidal erythrocytes with poorly-organized membrane lattice, decreases in height, peak-to-valley distances, and surface roughness 42. Surgical intervention, such as splenectomy, is a therapeutic strategy that has been adopted to relieve HS symptoms. A comparative study using AFM was performed to evaluate the efficacy of splenectomy on HS patients 42. Interestingly, although this surgical intervention was effective as a remedy for hemolytic anemia and other symptoms found in HS patients, AFM study revealed that there was no morphological restoration of erythrocytes, suggesting the need for a fundamental therapeutic intervention such as allogeneic hematopoietic stem cell transplantation. From the morphological point of view, the pathologic erythrocytes looking different from healthy ones were called elliptocytes appearing in the shape of ovals or elongated rods. AFM images obtained from erythrocytes in patients with iron deficiency anemia showed significant aggregation of membrane proteins. Further deformation was observed on the cell surface showing the swelling of the cell center deviated from the normal biconcave shape 43. From the study, complete restoration of the morphology of erythrocytes during treatment was proposed as a criterion to determine the appropriate time for treatment termination.

The most remarkable superiority of AFM images over other microscopic images is that they are capable of visualizing cellular morphology and the ultramicroscopic structures of the cell membrane. Traditional diagnosis of malaria relies on the microscopic examination of malaria parasitemia from stained blood samples smeared on glass slides 66. In this microscopic diagnosis, low resolution and dry conditions make it difficult to distinguish malaria parasites from similar species. Consequently, false-positive results, late detection, or absence of standardization in the diagnosis of malaria parasitemia have resulted in increased mortality 66,68,70,71. Epidemiological and molecular studies have reported that the spectrin-based cytoskeleton of erythrocytes is strongly associated with malaria pathogenesis 72,73. Spectrin is a cytoskeletal protein on the plasma membrane of erythrocytes, which forms a mesh structure by associating with actin filaments, and thus maintains plasma membrane integrity. During the progression of malaria pathogenesis, the erythrocytes infected by the human malaria parasites, *Plasmodium falciparum,* are expected to undergo substantial changes in membrane integrity and deformability for effective transmission to mosquitoes 74-76. Indeed, an AFM study confirmed the appearance of “knobs”, the assembly of adhesive proteins on the membrane of infected erythrocytes, the elongation of spectrin filaments, and the enlarged spectrin mesh during the progression from the ring (early) and trophozoite (growing) stages to the schizont (dividing) stages 77. Recent AFM study, involving coarse-grained molecular dynamics simulation, explicitly showed the reversible modulation of the spectrin-actin network 78.

An AFM study investigated changes in the morphology of host cells during viral infection. The virus entered the host cells through physical adhesion and engulfment to the host cells. The inevitable morphological and mechanical modulations of the host cells were anticipated during the viral infection. A significant protrusion and softening of the cell membrane, attributed to the viral infection, were directly revealed by the AFM topographic images 79. The study reported that the different sizes of membrane protrusion were associated with exocytosis of the protein structures and the progeny virus.

Despite the numerous advantages, AFM imaging technique is limited by time resolution; it usually takes about 5 min to obtain a single frame of image. To overcome this problem, high-speed atomic force microscopy (HS-AFM) was invented 80. The first generation of HS-AFM could capture the images of moving proteins at a rate of 80 ms per frame 80. With recent advances, the real-time imaging of biological process at a molecular level has been achieved. As an example, myosin V walking along actin filaments was successfully visualized by HS-AFM 81,82. Indeed, the fast-scan ability of HS-AFM seems very beneficial in the monitoring of the dynamic changes of biological specimens during pathological progression of diseases. An interesting study using HS-AFM has been reported by Watanabe-Nakayama *et al* 83. Using HS-AFM, they successfully monitored the dynamic process of fibril formation and elongation of amyloid β-protein (Aβ), a key pathogenic agent in neurodegenerative diseases such as Alzheimer disease. Amyloid fibril accumulation is associated with numerous neurodegenerative diseases 84-93. However, the mechanisms by which Aβ accumulation in the brain leads to neurodegeneration remain unclear. The clarification of the fibrillation mechanism, the structural features of the amyloid fibrils, and their physical and mechanical properties are expected to unveil the roles of amyloid fibrils in the progression of a range of conditions from mild cognitive impairments to Alzheimer's disease 94. HS-AFM images show two different growth modes of Aβ, one producing straight fibrils and the other producing spiral fibrils. The switch between two different growth modes was suggested to be a key step in determining Aβ polymorphisms associated with the pathogenic condition.

The AFM was also adapted to witness the effect of compounds or drugs inducing nanoscale morphological modification in single cells. For instances, glutaraldehyde is a common chemical fixative used to preserve cells/tissues for the electron microscopy. Shibata-Seki *et al.* investigated the effect of chemical fixation of glutaraldehyde on corneal endothelial cells using the AFM. They found that the treatment of glutaraldehyde resulted in shrinkage of the endothelial cells owing to the evaporation of water 95. Glycans play a key role in physiological and pathological processes by mediating cell-cell and cell-ECM interactions. Glycosylation of the biomaterials influences cell fate such as proliferation, differentiation, and functionality. Figuereido *et al.* used AFM to investigate the biocompatibility of neoglycosylated films on human SYSH-SY5Y neuroblastoma cell lines 96. The AFM topographic images showed that the neoglycosylated collagen films exhibited well-defined fibrillary structures, while untreated control had amorphous structures. Furthermore, the human SH-SY5Y neuroblastoma cells had comparable biocompatibility to the neoglycosylated collagen films. Their results suggested that the morphological alteration of the neoglycosylated collagen could modulate the inter-molecular and inter-fibrillar interactions of the triple-helical domain of the collagen films resulting in improved biological activity. Antimicrobial peptides are a promising class of antimicrobials that exhibits activity against antibiotic-resistant bacteria, parasites, and viruses. Fantner *et al.* used HS-AFM to monitor real-time morphological modification induced by the antimicrobial peptide CM15 on living *Escherichia coli* bacteria cells at a nanoscale resolution. They found that the cell surface changed from smooth to being corrugated after treating with CM15 97.

AFM has been used to monitor the morphological distortion of cells at nanometer resolution. The high-resolution images provided more quantitative and bias-free diagnostic means beyond the conventional diagnostic assays. Although the use of AFM imaging technique in the observation of biological samples has its own drawbacks, it offers an invaluable potential as a diagnostic tool especially when combined with existing diagnostic tools 98.

## Mechanical compliance

Many diseases are inherently accompanied by mechanical alteration of tissues and cells. For instance, skin aging induces decrease in skin resilience, which is attributed to changes in the composition and organization of extracellular matrix 99. Consequently, the mechanical properties of cells and tissues are important indicators of pathologic progression of diseases 100. Mechanical compliance, also called softness, indicates the flexibility of tissues or cellular materials under external stress. The extent of mechanical compliance is often expressed as elastic moduli, *i.e.,* Young's moduli, which can be calculated from stress-strain relation. A high Young's modulus indicates a low mechanical compliance 101. Over the past decades, various techniques have been developed to investigate the mechanical compliances of biological samples such as cells and tissues. These techniques include optical stretcher 102-105, micropipette aspiration 106, microfluidics 107, magnetic tweezers 108-110, and AFM 111-114. Among these, AFM has been the most widely utilized in bio-mechanical assays 13,52,115-118.

A simple indentation experiment was carried out to determine the elastic moduli of samples. Some attempts were made to obtain both the storage and loss moduli of biological samples by superimposing the oscillating motion on the probe while indenting the samples 113,115,119. For the clinical applications of AFM techniques, there have been an increasing number of studies investigating tissues obtained from a minimally invasive surgical intervention such as biopsy 47,50,120. One of the long-standing problems associated with probing tissues, in comparison with cells, is the technical difficulty associated with immobilizing tissue samples on hard substrates in liquid environments. To overcome this problem, a novel method was adopted for the efficient immobilization of tissues. Nanopillars of “bed of nails”-like approach to anchor pancreatic islet demonstrates an exemplary strategy to achieve proper immobilization of tissues 50. In addition, comparative studies that evaluated changes in elastic moduli with regards to storage and buffer conditions have been carried out to establish the standard conditions for AFM nano-mechanical studies on tissues 47.

Table 2 summarizes AFM-based bio-mechanical reports on changes in elastic moduli attributed to disease onset and progression. Interestingly, while samples from diseases such as sickle cell disease and cardiovascular complications showed increase in Young's moduli compared to healthy samples, other samples from diseases such as asthma, osteoarthritis, and diabetes conversely showed reduced mechanical integrity.

Sickle cell disease is an inherited disorder caused by genetic mutation in the hemoglobin. AFM indentation experiments performed on human erythrocytes harvested from patients with sickle cell disease showed that pathological erythrocytes is about three times stiffer than normal cells 44. The seven stranded polymer structure of hemoglobin S and changes in the affinity of spectrin and actin filaments were suggested to be responsible for the increased stiffness of sickled erythrocytes.

Mechanical stiffening has also been noted as a hallmark of cardiovascular complications 119,121. A recent AFM study directly observed a significant increase in the elastic moduli of ventricular tissues freshly harvested from mice with pressure overload-induced cardiac hypertrophy 121. Cardiac hypertrophy, a leading cause of cardiac complication-induced death, is characterized by the abnormal enlargement and thickening of the myocardium. The study also reported that increase in the elastic moduli of hypertrophic myocardium enhances the production of vascular endothelial growth factor through PI3K/Akt signaling pathway, thus facilitating angiogenesis during the progression of cardiac hypertrophy to heart failure. The mechanical stimuli from the stiffened matrix were found to be mediated by talin 1 and integrin β1. The findings of the study provided information not only on the direct quantification of mechanical changes in the myocardium but also on novel pharmacological interventions to slow down the detrimental progress of cardiac hypertrophy. Moreover, age-related aortic stiffening, another cause of heart failure, was quantitatively evaluated by an AFM-based bio-mechanical assay 119. The study was unique because frequency modulated atomic force microscopy (FM-AFM) was used to determine both the storage and loss moduli in localized regions. The study reported an age-induced elevation in elastic moduli, which was more prominent in the inter-lamellar regions than in the lamellar regions. Inter-lamellar regions are composed of complex meshwork of collagen fibers, elastin fibers, and smooth muscle cells, of which major rearrangements occurred due to aging, and thus their mechanical alterations were more severe than those of other regions.

Age-related mechanical degradation of human chondrocytes has been reported by an AFM study 48. In this study, it was observed that old chondrocytes had three-fold lower stiffness than normal counterparts. There was a similar observation with sodium nitroprusside (SNP)-induced chondrocyte apoptosis, a typical osteoarthritis model; SNP-treated chondrocytes showed remarkable decrease (90%) in elasticity 49. Chondrocytes are embedded in the extracellular matrix which is composed of collagens, proteoglycans, and glycoproteins to form articular cartilage. The aging of the articular cartilage results in cartilaginous degeneration such as osteoarthritis 122. The mechanical disintegration observed in old chondrocytes is strongly associated with distorted macromolecular framework attributed to the aging process, leading to the damage or death of chondrocytes 123,124. Strikingly, the mechanical disintegration of aged chondrocytes is seemingly contrary to the age-dependent mechanical modulation of human articular cartilage. Stolz *et al.* reported the age-dependent-stiffening of articular cartilage with progressive decrease in glycosaminoglycan contents 125. However, the elastic moduli observed in osteoarthritis patients confirmed the progressive softening of articular cartilage. The age-dependent stiffening of human articular cartilage is overruled by the progressive softening found in osteoarthritis. Again, the mechanical softening of articular cartilage is attributed to the disintegration of collagen meshwork. In addition, such mechanical modulation of articular cartilage was apparent not at micrometers but at nanometers of indentation depth. This requirement of nano-manipulation suggests that the AFM-based indentation technique might serve as a pre-symptomatic diagnostic tool for osteoarthritis. A study has demonstrated that the elastic modulus determined by AFM indentation experiments can be utilized as a progressive disease marker during the treatment of osteoarthritis 49. The study also reported the preventive effect of resveratrol on SNP-treated chondrocytes. Resveratrol is a polyphenol, derived from some fruits such as grapes, and an anti-inflammatory agent 49. The elastic moduli, measured by AFM, showed that pretreatment with resveratrol prevents chondrocytes from undergoing SNP-induced mechanical disintegration. Immunofluorescent images revealed that such mechanical modulations resulted from the active reorganization of the actin cytoskeleton induced by resveratrol treatment.

It is also fascinating that AFM studies have also reported close correlation between inflammatory diseases, such as asthma, and the mechanical softening of tissues 47,50. The mechanosensitive production of insulin from islets was confirmed by AFM-based nano-indentation experiments performed on transgenic DORmO mouse model of type 1 diabetes 50. The study showed that autoimmune insulitis resulted in mechanically soft islets. The intraislet accumulation of hyaluronan prior to the onset of diabetes was considered as a major cause of such mechanical changes. Hyaluronan, a polymer in the extracellular matrix is highly hygroscopic, and thus, its increased accumulation promotes hydration and softening of tissues. Asthma is a hyper-responsive complication in the airway characterized by chronic inflammation. Structural remodeling of bronchial walls includes aberration in the extracellular matrix composition, increase in collagen type I, III, and V, as well as fibronectin, and decrease in collagen type IV 126,127. Lately, AFM studies on tissues from bronchial biopsies reported lower elastic moduli in airway tissues collected from asthma patients than those in tissues collected from healthy volunteers 47. Although the major determinants contributing to the reduced mechanical stiffness of bronchial tissues in asthmatic patients are yet to be determined, AFM nano-indentation has emerged as an early and quantitative diagnostic tool for asthma, a respiratory complication with various symptoms.

Remarkably, an AFM nano-indentation study has been conducted to investigate fundamental information required to fight against intractable diseases such as human immunodeficiency virus (HIV) infections and nerve injury. The study provided quantitative evidences to depict the mechanically switching behavior of HIV during the infection process 128. A stunning switch between softening and stiffening behaviors took place from viral budding to viral entry into the host cells. More detailed investigation was conducted to address the mechanical stability of HIV-1 capsid using AFM, revealing the mechanical hardening of hyperstable mutants. Mutations modulating capsid stability are known to largely affect HIV infectivity. Similarly, AFM experiments were carried out to depict the fundamental mechanism of axonal degeneration due to nerve injury or compression. A state-of-the-art study combining microfluidics with AFM was performed to determine the threshold force required to compromise axonal survival after compression 129. The study showed that rat hippocampal axons fully recovered axonal transport with no detectable axonal loss when compressed with pressure up to 65 ± 30 Pa for 10 min. Whereas, the dorsal root ganglia axons, which showed 20% lower elastic modulus than hippocampal axons, resisted pressure up to 540 ± 200 Pa. It was suggested that the integrity of axonal cytoskeleton mainly affects axonal fate after damage. AFM-based force spectroscopy, combined with fluorescence microscopy, has also been used to determine changes in neuronal stiffness during neurite outgrowth 130. Fluorescence images indicated that the organization of microtubules is a major component associated with neuronal stiffness. In addition, the AFM study explicitly determined that the growth cones of axotomized neurons underwent mechanical softening during sciatic nerve injury 131. Although it is too early to determine if AFM demonstrates diagnostic advantages for HIV and nerve injury, the obtained information from AFM studies would be crucial to the improvement of pharmacological interventions for the treatment or prevention of these intractable diseases.

Each of the aforementioned diseases has its own underlying mechanism that causes the corresponding mechanical alterations. Nevertheless, each disease generally involves the aberrant reorganization of actin cytoskeleton or the extracellular matrix. Depending on the extent of hydration, the organization and composition of the extracellular matrix or the actin cytoskeletal proteins are modulated during disease onset and progression, and changes in elastic moduli appear in two different directions of the stiffness spectrum as shown in Table 2. Even the reversible switching behavior was observed in HIV infection. As shown in osteoarthritis, the interplay between mechanical changes in cells and their microenvironments collectively affect the mechanical behavior of tissues during disease progression. Herein, we show that AFM-based biomechanical studies are promising in the early diagnosis of diseases because they are able to detect the pre-symptomatic changes in the mechanical properties of cells and tissues in many pathological conditions.

## Adhesion properties

Cell adhesion plays an important role in cell communication and regulation. The mechanical interaction between a cell and its extracellular matrix (ECM) controls cellular behavior and functions. The alteration of cell adhesion can be a defining event for the onset of numerous diseases such as type 2 diabetes, neurodegenerative diseases, osteoarthritis, cardiovascular diseases and sickle cell anemia 132-136. Tremendous efforts have been made to develop various techniques to quantitatively determine cell adhesion 137. Recently, AFM has been extensively utilized to determine the adhesive properties of cells 31,135,136,138-141.

Simple studies using the AFM-based adhesion assay were performed to investigate bacterial adhesion on dental surfaces 28,142. Bacterial adhesion is considered as a primary cause of periodontal diseases such as dental caries and cavities 143. For more than half a century, the fluoride treatments of teeth have been carried out in order to prevent dental caries. An AFM study revealed that the reduced bacterial adhesion on enamel surfaces is a key factor associated with the cariostatic effect of fluoride treatment 142. Furthermore, dental restorative materials such as composite resin Amelogen® and dental alloy often attract bacterial adhesions, resulting in the formation of secondary caries. AFM-based force spectroscopy evaluated the adhesion forces of cariogenic pathogens such as *Staphylococcus aureus* on dental restorative materials as shown in Table 3. The finding of the study suggested that surface roughness and free energy on initial staphylococcal adhesion forces are the main characteristics to be considered for dental restorative materials.

As summarized in Table 3, AFM-based adhesion assays investigate how various pathologic conditions lead to changes in the adhesion properties of cells. First, it has been shown that aging and diabetes increase the adhesion properties of erythrocytes 45. Type 2 diabetes is a metabolic disorder with high sugar levels in the blood owing to insulin resistance or deficiency 144-146. It has been postulated that high level of glucose in the blood enhances viscosity and aggregation in the membrane of erythrocytes. The increased adhesion properties of the erythrocytes of old people explains why elderly persons are more vulnerable to vascular diseases including diabetes. Remarkably, the increase in the adhesion properties of erythrocytes found in patients with type 2 diabetes were more significant than the changes observed in old healthy people.

Unlike the diabetes-induced changes in adhesion properties, some diseases such as osteoarthritis lead to decrease in the adhesion properties of cells 48. Clinically, osteoarthritis (OA) is diagnosed with radiography 147. Radiographic examination provides information on bony changes that occur during OA prognoses such as osteophyte formation, subchondral sclerosis, asymmetric joint space narrowing, subchondral cysts, and subluxation 147-151. However, radiography diagnosis often provide a delayed or missed diagnosis of OA 152. Thus, an AFM-based study was performed to investigate the prognoses of OA 48. The study showed that the adhesion forces of OA chondrocytes were relatively low and distributed over a narrow range compared to normal chondrocytes (see Table 3). Furthermore, the study revealed a decrease in integrin β_1_ mediated chondrocyte-ECM interactions in OA, implicating the perturbation of cell matrix in OA. The down-regulated expression of integrin β_1_ in OA chondrocytes was observed as the main mechanism behind the reduced adhesion forces of OA chondrocytes.

The adhesions detected by bare AFM probe involve the collective interactions between the probe and various proteins on the cell membrane. It is not easy to determine which component among the various adhesion-mediating molecules is responsible for such modulated adhesions observed in diabetes and OA. There were attempts to adopt AFM-based single-molecule force spectroscopy (SMFS) in order to determine specific molecules mediating adhesions in pathologic conditions. The AFM-based SMFS detects single functional receptors on cells and the unbinding force between a receptor and the corresponding ligand. Several pathologic disorders such as sickle cell disease, inflammation, and autoimmune blistering skin disease were investigated using this AFM technique. The sickled red blood cells (RBCs) are known to show increase in adhesions to other RBCs and the endothelium. The enhanced adhesion of RBCs to the endothelium causes a delayed microvascular passage of deoxygenated RBCs, promoting sickling and entrapment of RBCs. Consequently, these series of events initiate vaso-occlusive episodes, which are characteristics of sickle cell disease. An AFM-based adhesion assay identified that increase in the binding events between intercellular adhesion molecule-4 (ICAM-4) and α_v_β_3_ integrin results in the abnormal adhesion of sickled RBCs to endothelial cells 27. The study also showed that ICAM-4 is activated by cyclic adenosine monophosphate-protein kinase A-dependent pathway 27. The recruitment of leukocytes into injured tissues leads to the progression of inflammation 153. In the beginning of inflammatory pathogenesis, leukocytes in the blood circulation adhere to vascular endothelial cells and migrate through endothelial cells into the interstitial space. Jaczewska *et al.* showed that the adhesion of Jurkat cells to stimulated HUVEC monolayers took place remarkably in junctional regions 154. Furthermore, AFM adhesion mapping revealed that the redistribution of junctional adhesion molecule-A (JAM-A) along junctional regions plays a key role in mediating lymphocyte recruitment to the endothelium and subsequent transendothelial migration under inflammatory conditions. AFM adhesion study was also utilized to investigate the molecular signature of gap junctions associated with autoimmune blistering skin disease such as pemphigus vulgaris (PV) 155. Desmosomal junctions are cadherin-based intercellular junctions in epithelial tissues and their disruption strongly correlates with the incidence of PV. The study revealed that the incubation of pathogenic antibodies with desmoglein 3 resulted in the disruption of intercellular adhesion and structural changes in human keratinocytes, leading to blister formation.

Furthermore, AFM-based adhesion study has been used to determine the effects of trace elements on the progression of neurodegenerative diseases. With trace metals such as copper and zinc, the aggregation and neurotoxicity of amyloid-β (Aβ) is significantly enhanced. The aggregation of Aβ is considered as a major cause of Alzheimer's disease. Recently, Hane *et al.* used AFM to characterize the kinetic and thermodynamic parameters of the dissociation of an Aβ dimer in the presence of copper and zinc ions 156. Their results demonstrated that while copper at a nanomolar concentration did not alter the single molecule affinity of Aβ-Aβ, zinc at the nanomolar concentration reduced the Aβ-Aβ affinity.

Overall, different studies have indicated that altered cell adhesion is a defining event in the onset and progression of various pathological conditions. The accurate determination of cell adhesion is critical information for the diagnosis and prognosis of diseases. We believe that molecular signatures revealed by AFM-based force spectroscopy provide valuable information not only for the diagnosis of different diseases but also for the development of therapeutic strategies.

## Molecular recognition imaging

The cell membrane is composed of interdependent species of molecules, molecular groupings, and supramolecular entities, which play a crucial role in cell functions such as cell adhesion, cellular communication, tissue development, inflammation, tumor metastasis, and microbial infection 1,157. Some molecules act as receptors and others as sensors controlling the important cellular processes 158,159. The malfunction of membrane proteins often results in the onset of diseases. Many diseases including pseudoexfoliation 34, cystic fibrosis 35, neurodegenerative diseases 37, and pertussis 36 are associated with alterations in the molecular composition of cell membrane. Consequently, many therapeutic drugs target the human membrane proteins 160. Previously, cryo-electron microscopy 161, photoactivated localization microscopy 162, and X-ray crystallography 163 have been used to obtain the molecular structures of the cell membrane. Nevertheless, the images obtained from these techniques provided distorted information due to the pretreatment of the target molecules 2. Thus, the emergence of AFM offers an exciting methodology to monitor membrane proteins in near-native physiological conditions.

Molecular recognition imaging technique using AFM combines molecular recognition with force microscopy 32,164. The obtained images successfully show the chemical composition of the sample as well as its topographical structures. This technique allows molecular recognition with concentrations below the detection limits of current technologies such as enzyme-linked immunosorbent assay, mass spectroscopy, and protein microarray 165, 166. In order for AFM to detect specific targets, the AFM probe has to be functionalized with a molecule of high affinity 33,167. The force applied to break the chemical bond between the probe and the target molecule is quantitatively determined from the force-distance curve 168-170. Previous studies have demonstrated that a silicon nitride cantilever tip functionalized with DNA aptamers and cyclo-RGD peptides is able to detect their cognate *α-*thrombin, IgE molecules, and integrin α_5_β_1_ with high accuracy upto ~90 % 33,171.

The cystic fibrosis transmembrane conductance regulator (CFTR) is a channel localized on the apical membrane of the epithelial cells lining exocrine glands. The CFTR maintains the salt and water balance in the epithelium and regulates cell volume 172,173. CFTR dysfunction results in a severe disease known as cystic fibrosis (CF), characterized by impaired epithelial transport in the respiratory system, liver, and pancreas. The prevalent alteration is the deletion of the amino acid phenylalanine at position 508, subsequent misfolding, impaired trafficking to the membrane, and the reduced number of CFTR on the plasma membrane 174-176. Clinically, a sweat test is used to measure the level of the chloride ions in sweat via quantitative coulometric test or chloride titration test. However, the sweat test method is highly unreliable because of insufficient production of sweat 177. Ebner *et al.* used the AFM molecular recognition imaging technique to investigate CFTR in erythrocytes membrane at the single-molecule level 46. While the normal human erythrocytes have high permeability to chloride 178,179, the erythrocytes of CF patients with the F508del mutation showed about 30 % decrease in CFTR on the plasma membrane.

The AFM molecular recognition imaging technique was also adapted for the monitoring of protein aggregation on the human lens capsule 180. Pseudoexfoliation syndrome (PEX) is charaterized by the deposition of whitish-grey extracellular fibrins in the anterior lens capsule, leading to irreversible blindness 181-183. PEX is usually diagnosed by slit lamp using biomicroscopy 184. However, biomicroscopy technique cannot provide information on protein changes that cause the onset of PEX, to facilitate the development of treatment modalities. The AFM molecular recognition images obtained by Creasey *et al.* identified the localized distribution of clusterin, one of the proteins implicated in PEX, while indicating that clusterin did not follow a specific distribution pattern observed on normal lens capsules 51. The distribution pattern occurred due to the aggregation of misfolded proteins in PEX, leading to a chaperone response by clusterin. This investigation shows the feasibility of the use of AFM molecular recognition imaging technique to detect pathological alteration of biological tissues.

Interestingly, the nicotinic acetylcholine receptor (nAChR) on neurons from the ventral respiratory group was monitored by AFM molecular recognition imaging technique 185. nAChR is a member of the ligand-gated ion channels in the central and peripheral nervous systems 186. The functional alteration in the α7 nAChRs has been implicated in the abnormal function of cells such as cell replication and differentiation, axonogenesis/synaptogenesis, as well as synaptic function and behavior 186, 187. AFM probe was conjugated with anti-α7 subunit nAChR antibody, which interacts with the surface of NK1-R positive neurons. Acute exposure to nicotine caused an 80% decrease in the binding ability of nAChR antibody to the α-7 subunit in a dose-dependent manner. The study suggested that nicotine exposure reduced the binding probability of α-7 subunit-containing nAChRs, which correlated with the loss of nicotinic receptor function.

AFM molecular recognition technique has been extended into the study of medically important microbes. Pertussis is a toxin-mediated disease; *Bordetella pertussis* attaches to the cilia of the respiratory epithelial cells and secrets exotoxin. The secreted exotoxin incapacitates the cilia and results in the inflammation of the respiratory tract, which interferes with the clearing of the pulmonary secretion 188. Bacteria adhesion is the most significant step in the development of bacteria infection. Pertactin, fimbriae, and filamentous haemagglutinin adhesin (FHA) proteins interact with different components of the respiratory epithelium to facilitate the attachments of the cells. FHA participates in the first step of adhesion through recognition domains by adhering to respiratory epithelial cells and macrophages 188-190. In the clinical field, polymerase chain reaction is used for the detection of DNA sequences of *B. pertussis* 191. AFM-based force spectroscopy assay was performed to obtain information about the localization and distribution of FHA-mediated adhesions in *B. pertussis* 192. The force strength of the recognition events ranged from 50 pN to 900 pN. Cluster and nearest neighbor analysis revealed that the amount of cluster diminished during the time-lapsed imaging but the size of connected clusters markedly increased. It was shown that the active clustering of bacterium nanodomains on the cell membrane is a crucial step in bacterial infection. The study successfully demonstrated the application of AFM-based molecular recognition imaging technique in monitoring the spatio-temporal rearrangements of adhesins at the molecular level. The obtained information might contribute to understanding the basic molecular mechanisms through which bacterial pathogens cause infectious diseases.

Here, we understand that AFM molecular recognition imaging technique is widely utilized in the imaging of biological specimens including cells, tissues, and bacteria. It is demonstrated that this technique is capable of nanoscale imaging resolution and time-lapsed imaging ability. In addition, AFM provides highly reproducible, specific, and efficient molecular detection, and consequently, alteration in tissue and cell structures under pathological condition can be closely monitored. The detection of antigens is also more efficient without the lengthy preparation procedures. We anticipate that molecular recognition techniques would be very useful in the development of therapeutic modalities for various diseases.

## Conclusion

In this review, we address the basic techniques of AFM, which have been widely utilized in the detection of pathological conditions. The increasing number of studies adapting AFM as a vital tool in the study of various pathological conditions indicates the intense scientific awareness of its potential. The nanoscale imaging capability has made it possible to detect morphological changes associated with diseases such as hereditary spherocytosis, iron deficient anemia, malaria, and neurodegenerative diseases. Accurate determination of cell/tissue stiffness has improved the early diagnosis of diseases such as sickle cell anemia, asthma, type 1 diabetes, osteoarthritis, cardiovascular diseases, and HIV infection. AFM can also be utilized to quantify the adhesion properties of cells. Diseases such as type 2 diabetes, osteoarthritis, sickle cell diseases, inflammatory diseases, Alzheimer's diseases, and periodontal diseases have been monitored in terms of adhesion properties. The molecular recognition imaging technique has revolutionized exploration of biological specimens at a single molecule level. Remarkably, this technique is a unique force spectroscopic tool which enables us to monitor the spatial distribution of chemical heterogeneity with the nanometer precision. Specific single molecule interactions include an antigen-antibody and a ligand-cell surface receptor bond. These interactions are prevalent in numerous biological processes such as immune response, genome replication and transcription, and infection. Interestingly, molecular recognition imaging techniques, using antibodies and antigens of interest, expand the application of AFM in pathologic investigations with a high degree of sensitivity and specificity. Cystic fibrosis, pseudoexfoliation syndrome, neurodegenerative diseases, and whooping cough have been studied using AFM-based molecular recognition imaging techniques. We expect that the diverse aforementioned AFM techniques would make a tremendous improvement in clinical studies. The molecular information obtained from AFM would facilitate early diagnosis of diseases before they progress to complications which cannot be treated by the current therapeutic modalities.

## Figures and Tables

**Table 1:** Morphological changes observed by the AFM topography during disease onset and progression

Disease	Cells	Imaging condition	Changes from healthy to pathological conditions	Reference
Hereditary Spherocytosis	Human Erythrocytes	IC (Live cells)	Decrease in L from 6.51 ± 0.27 nm to 5.92 ± 0.27 nm and in W from 6.22 ± 0.15 nm to 5.81 ± 0.12 nm	42
			Increase in height from 586 ± 120 nm to 1,644 ± 34 nm	
			Decrease in Rp-v from 1,272 ± 48 nm to 1,058 ± 155 nm	
			Decrease in Ra from 287 ± 44 to 215 ± 6	
Iron deficiency anemia	Human Erythrocytes	CM (Dead cells)	Increase in protein particle size from 8 nm to > 140 nm	43
			Increase in r from 1.01 ± 0.06 to 1.52 ± 0.47	
			Decrease in H from 1,729 ± 39 nm to 1,518 ± 72 nm	
			Increase in h from 41 ± 6 nm to 403 ± 43 nm	
			Decrease in Rp-v from 1,605 ± 29 nm to 1,154 ± 88 nm	
			Increase in Ra from 582 ± 26 nm to 1,227 ± 91 nm	
Malaria	Cultured Erythrocytes	CM and IC (Dead cells)	Increase in spectrin length from 48 ± 7 nm at ring stage to 64 ± 9 nm at early and middle trophozoite stage, 69 ± 10 nm at late trophozoite stage, and 75 ± 11 nm at schizont stage	77
	Human red blood cells	IC (Dead cells)	Changes in spectrin length from 61 ± 14 nm at trophozoites stage, to 62 ± 8 nm at gametocytes stage, and 42 ± 12 nm at stage V gametocytes	78
Pox virus	Kidney cells	Live cells	Exocytosis of protein structures: 10-100 nm in diameter	79
			Exocytosis of progeny virus: 200-300 nm in diameter	
Neurodegenerative diseases	Aβ_1-42_ fibril	HSI	Observed two growth modes of Aβ_1-42_: One producing straight fibrils and another producing spiral fibrils	83

**Abbreviations:** Aβ _1-42_: amyloid β peptide; CM: contact mode; h: valley height; H: peak height; HSI: high-speed imaging; IC: intermittent Contact; L: length; Ra: surface roughness; Rp-v: peak-to-valley distance; r: ratio of length to width; W: width.

**Table 2:** Alteration in mechanical compliance of cells and tissues revealed by the AFM studies during disease progression

Pathological changes in E	Disease	Samples	Changes from healthy to pathological condition	References
Increase	Sickle cell disease	Human erythrocytes	1.10 ± 0.40 kPa to 3.0 ± 1.09 kPa	44
	Cardiac hypertrophy	Mice ventricular tissues	13.5 ± 0.665 kPa to 34.1 ± 1.37 kPa	121
	Aging	Sheep aorta	Lamellar region: Young (36 ± 2.22 Kpa) to old (63 ± 2.95 Kpa) Inter-lamellar region: Young (25 ± 3.39 Kpa) to old (63 ± 2.76 kPa)	119
	Aging	Mice articular cartilage	23 ± 1.9 kPa at 6 months to 41 ± 2.9 kPa at 12 months	125
Decrease	Osteoarthritis	Human chondrocytes	0.0960 ± 0.009 N/m to 0.0347 ± 0.005 N/m	48
		Human articular cartilage	83 Kpa to 5.6 kPa	125
		Mice articular cartilage	38 ± 3.4 kPa at 6 months to 20 ± 3.4 kPa at 12 months	
		Rabbit chondrocytes	1.43 ± 0.45 Mpa to 0.16 ± 0.08 Mpa	49
	Type 1 diabetes	Mice islets tissue	∼3 kPa to ∼284 Pa	50
	Asthma	Human bronchial tissue	Lower stress: 14.6 ± 8.2 kPa to 7.7 ± 4.0 kPa	47
			Higher stress: 3.5 ± 1.8 kPa to 1.8 ± 1.0 kPa	
	HIV-1 virus	Virus particles	Immature HIV-1 virus: 0.93 GPa	128
			Mature HIV-1 virus: 0.44 GPa	
	Nerve injury	Mice neurons	Softening of growth cone about 20 to 40 %	131

**Abbreviation:** E: elastic modulus.

**Table 3:** Changes in adhesion properties revealed by the AFM studies during disease progression

Disease	Sample	Functionalization on the probe	Changes from healthy to pathological conditions	References
Type 2 diabetes	Human erythrocyte	None	Increase in AF from 200 ± 38 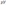 (young) and 420 ± 25 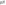 (old) to 510 ± 63 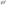	45
Osteoarthritis	Human articular chondrocytes	None	Decrease in AF from 7 ± 3 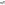 to 2 ± 1 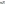	48
Sickle cell diseases	Human red blood cells	Integrin *α*v*β*3	Increase in ICAM-4 AF after epinephrine treatment from 10.08 ± 0.85 % to 21.41 ± 2.27 %	27
Inflammatory diseases	Human T-leukemia Jurkat cells and HUVECs	Human junctional adhesion molecule-A antibody	Increase in DA of stimulated HUVEC center from 1 × 10^-16^ ± 0.42 J to 3 × 10^-16^ ± 0.55 J and junction from 1 × 10^-16^ ± 0.45 J to 7 × 10^-16^ ± 0.58 J	154
Alzheimer's disease	Cys-Aβ42	Aβ peptides	Increase in Aβ-Aβ affinity from 0.40 ± 0.16 mM without zinc to 7.50 ± 2.06 mM with zinc ions	156
Periodontal diseases	Human whole saliva and *Staphylococcus aureus* bacteria	*Staphylococcus aureus* bacteria	Cobalt-nickel-chromium for dental alloy AF: 5.9 nN	28
			Feldspathic ceramic AF: 7.7 nN	
			Composite resin Amelogen^®^ Plus AF: 7.8 nN	
			Denture base polymer for dental prosthesis AF: 11.6 nN	

**Abbreviations:** AF: adhesive force; DA: de-adhesion force; HUVEC: human umbilical vein endothelial cell; ICAM-4: intercellular adhesion molecule-4.

## Abbreviations

Aβ: amyloid β-protein
Aβ _1-42_: amyloid β peptide
AF: adhesive force
AFM: atomic force microscopy
CF: cystic fibrosis
CFTR: cystic fibrosis transmembrane conductance regulator
CM: contact mode
DA: de-adhesion force
E: elastic modulus
ECM: extracellular matrix
FHA: filamentous haemagglutinin adhesin
FM-AFM: frequency-modulated atomic force microscopy
h: valley height
H: peak height
HIV: human immunodeficiency virus
HS: hereditary spherocytosis
HSI: high-speed imaging
HUVEC: human umbilical vein endothelial cell
IC: intermittent contact
ICAM-1: intracellular adhesion molecule 1
ICAM-4: intercellular adhesion molecule-4
JAM-A: junctional adhesion molecule-A
L: length
nAChR: nicotinic acetylcholine receptor
OA: osteoarthritis
PEX: pseudoexfoliation syndrome
PV: pemphigus vulgaris
R: ratio of length to width
Ra: surface roughness
RBCs: red blood cells
Rp-v: peak-to-valley distance
SMFS: single-molecule force spectroscopy
SNP: sodium nitroprusside
VCAM-1: vascular cell adhesion molecule-1
VLA-4: very late antigen-4 integrin
W: width
